# Exploring the mTOR Signalling Pathway and Its Inhibitory Scope in Cancer

**DOI:** 10.3390/ph16071004

**Published:** 2023-07-14

**Authors:** Suhail Ahmad Mir, Ashraf Dar, Saad Ali Alshehri, Shadma Wahab, Laraibah Hamid, Mohammad Ali Abdullah Almoyad, Tabasum Ali, Ghulam Nabi Bader

**Affiliations:** 1 Department of Pharmaceutical Sciences, University of Kashmir, Hazratbal, Srinagar 190006, Jammu and Kashmir, India; 2 Department of Biochemistry, University of Kashmir, Hazratbal, Srinagar 190006, Jammu and Kashmir, India; 3 Department of Pharmacognosy, College of Pharmacy, King Khalid University, Abha 62529, Saudi Arabia; 4 Department of Zoology, University of Kashmir, Hazratbal, Srinagar 190006, Jammu and Kashmir, India; 5 Department of Basic Medical Sciences, College of Applied Medical Sciences in Khamis Mushyt, King Khalid University, Abha 61412, Saudi Arabia

**Keywords:** cancer, regulation of mTOR signalling pathway, mTORC1/2, mTOR inhibitors, rapamycin

## Abstract

Mechanistic target of rapamycin (mTOR) is a protein kinase that regulates cellular growth, development, survival, and metabolism through integration of diverse extracellular and intracellular stimuli. Additionally, mTOR is involved in interplay of signalling pathways that regulate apoptosis and autophagy. In cells, mTOR is assembled into two complexes, mTORC1 and mTORC2. While mTORC1 is regulated by energy consumption, protein intake, mechanical stimuli, and growth factors, mTORC2 is regulated by insulin-like growth factor-1 receptor (IGF-1R), and epidermal growth factor receptor (EGFR). mTOR signalling pathways are considered the hallmark in cancer due to their dysregulation in approximately 70% of cancers. Through downstream regulators, ribosomal protein S6 kinase β-1 (S6K1) and eukaryotic translation initiation factor 4E binding protein 1 (4E-BP1), mTORC1 influences various anabolic and catabolic processes in the cell. In recent years, several mTOR inhibitors have been developed with the aim of treating different cancers. In this review, we will explore the current developments in the mTOR signalling pathway and its importance for being targeted by various inhibitors in anti-cancer therapeutics.

## 1. Introduction

**Figure 1:**
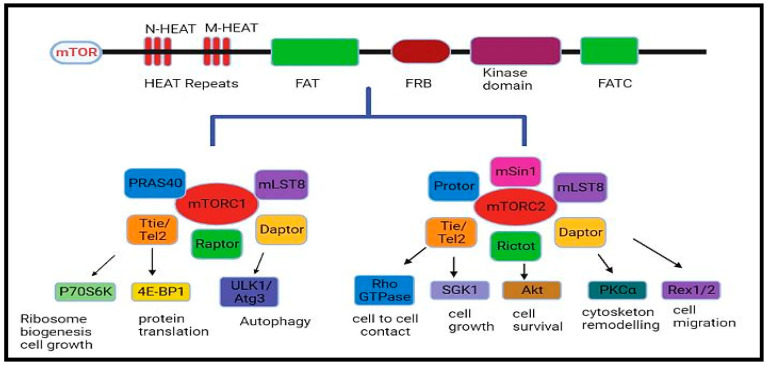
The mTOR complex, mTORC1, and mTORC2.

The mammalian target of rapamycin or mechanistic target of rapamycin (mTOR) is a serine–threonine protein kinase that modulates cellular metabolism, growth, cell proliferation or survival and gene expression phenomena [1]. Structurally, mTOR is a 289-kDa protein that is expressed in almost all the tissues of the body [2]. The phosphorylation of mTOR is triggered at particular residues (S1261, T2446, S2448, S2481) in response to growth factors, mitogens, hormones, and nutrition, whereas a lack of these factors and oxygen can inhibit its activation and enzymatic activity [3]. There are two complexes of mTOR, mTORC1 and mTORC2, which are physically and functionally distinct. While mTORC1 is comprised of mTOR, GβL (positive regulator of mTOR, which increases the kinase activity of mTOR), deptor, and raptor, mTORC2 is comprised of rictor, mTOR, proline rich 5 (PPR5), GβL, deptor and SIN1 (Figure 1). The role of various components of this signalling pathway is summarized briefly in Table 1 [4]. mTOR is linked to many signalling cascades, including phosphoinositide 3-kinase/Protein kinase-B, also known as Akt (PI3K/Akt), LKB1 (Liver kinase B-1/adenosine 5′-monophosphate-activated protein kinase (AMPK), tuberous sclerosis complex subunit 1 TSC1/TSC2/Ras homolog enriched in brain (Rheb), and Vam6/Rag GTPases [5]. It regulates cell proliferation, apoptosis, and autophagy (autophagic cell death) pathways by influencing various key processes like transcription and protein synthesis by integrating diverse signalling stimuli [6].

mTOR signalling is implicated in various diseases, like cancer, arthritis, insulin resistance, and osteoporosis and in tumour angiogenesis [7]. Several reports support that there is an aberrant activation of the mTOR signalling pathway in cancer [8,9]. Keeping in view the role of this important signalling cascade in crucial stages of cancer development, this review is aimed at highlighting the regulation of mTOR signalling pathway and its involvement in tumourigenesis. Furthermore, the latest findings on mTOR inhibitors in the treatment of various cancers are discussed.

**Table 1:** The various components of the mTOR complex, and their functions.

mTOR Components	Associated with	Mode of Action	Reference
Raptor	mTORC1	Positive regulator	[10]
Rictor	mTORC2	Positive regulator	[11]
Daptor	mTORC1 and mTORC2	Negative regulator	[12]
Ttie/Tel2	mTORC1 and mTORC2	Positive regulator	[13]
Protor	mTORC2	Positive regulator	[14]
mLST8	mTORC1 and mTORC2	Positive regulator	[15]
PRAS40	mTORC1	Negative regulator	[16]
mSin1	mTORC2	Positive regulator	[17]

## 2. Assembly of mTOR Complex

**Figure 2:**
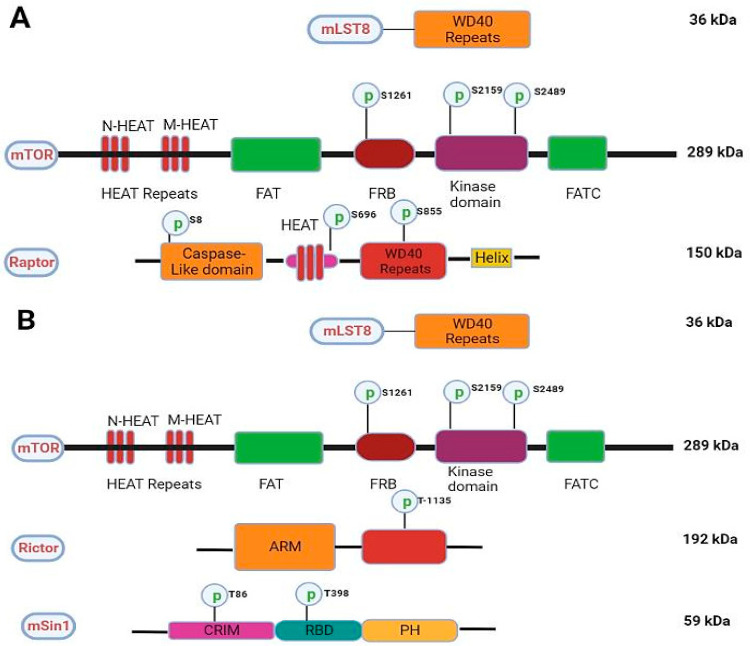
Schematics of the mTORC1 and mTORC2. (**A**) showing various domains, phosphorylation sites, and molecular weights in key components of mTORC1. (**B**) showing various domains, phosphorylation sites, and molecular weights in key components of mTORC2.

The mTOR protein is made up of 2549 amino acids and is organized into many domains, like NH2-terminal (N-HEAT), middle HEAT (M-HEAT), FAT domain (also called FKBP12-rapamycin-associated protein, ataxia-telangiectasia and transactivation/transformation), kinase domain with FRB (FKBP12 rapamycin-binding domain site) and a FAT carboxy-terminal domain. [18]. To form a dimer under physiological conditions, the HEAT repeats (12–13) of one mTOR monomer interact with the HEAT repeats (20–23) of another mTOR’s M-HEAT domain as illustrated in Figure 2A,B [19]. Because its C-terminal region shares considerable homology with the catalytic domain of PI3K, mTOR is also considered a member of the phosphatidylinositol-3-kinase superfamily [20]. Earlier, the difference between mTORC1 and mTORC2 was linked to their definite elements and susceptibility to rapamycin that selectively suppresses its activation of the mTORC1/2 complex. Originally it was thought that mTORC1 was rapamycin-sensitive, whereas mTORC2 does not bind to the drug. However, it is now a well-established fact that chronic exposure to rapamycin inhibits both complexes [21]. mTOR, DEP domain-containing mTOR interacting protein (Deptor), Ttie/Tel2 complex, and mammalian lethal with sec-13 protein 8 (mLst8) are the common subunits present in both mTORC1 and mTORC2 [22].

## 3. Regulation of mTORC1

**Figure 3:**
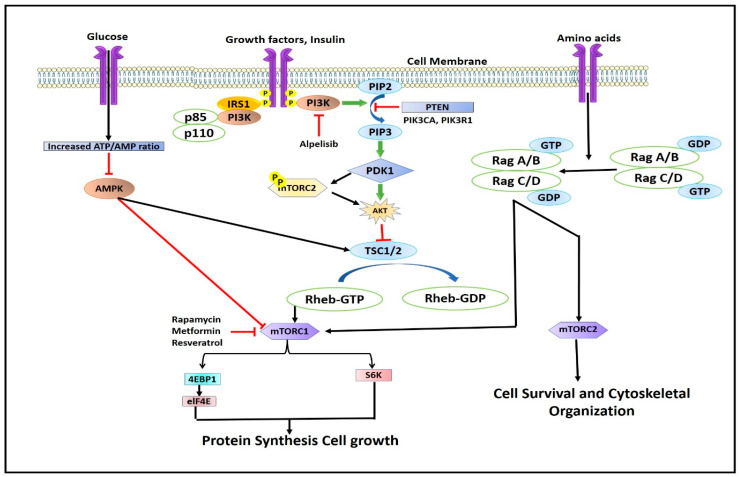
Illustration describing the regulation of mTOR complex.

Although mTOR exists in two separate complexes, there exists a cross-talk between mTORC1 and mTORC2. mTORC2 upregulates mTORC1 by activating the IGF-IR-Akt axis [1,23]. mTORC1 inhibits mTORC2 by phosphorylating mSin1 and Rictor on T86/398 and T1135 through its downstream regulator S6K1 via a feedback mechanism [24,25]. The mTORC1 complex is regulated by energy consumption, protein intake, mechanical stimuli, and growth factors (Figure 3). All of these components transmit signals to cells, which are subsequently detected, transformed, and then integrated together, causing cellular functions to alter. Signals are then detected by proteins containing surface receptors and intracellular kinases [26,27]. mTORC1 is mainly regulated by insulin-like growth factor-1 (IGF-1R) and insulin receptor (IR), which are tyrosine kinase receptors whose activation leads to the phosphorylation of insulin receptor substrates [27]. The insulin receptor substrates (IRS) bind and activate Src Homology 2 (SH_2_) domain-containing phosphatidylinositol-3-kinase, which in turn phosphorylates inositol phospholipids and forms phosphatidylinositol (3,4,5)-triphosphate (PIP_3_). The PIP_3_ then interacts with the Pleckstrin homology domain (PH) containing proteins like Akt and 3-phosphoinositide-dependent kinase (PDK_1_) [28]. The interaction of PIP_3_ with Akt phosphorylates and activates PDK_1_. Akt, which is expressed in skeletal muscles, is the main and important upstream regulator of mTORC1. Akt is activated by phosphorylation at serine residue Thr308 by PKD_1_ [29,30,31]. Active AKT phosphorylates tuberous sclerosis complex-2 (TSC2) leading to its suppression. TSC2 complex is a GTPase-activating protein (GAP) complex, which is comprised of TSC1/2 and TRE2-BUB2-CDC1_6_ domain family member 7 (TBC_1_D7) [32,33]. Akt activates Ras homolog enriched in the brain, Rheb, a GTP-binding protein, which leads to the activation of mTORC1 [34]. At the lysosomal membrane, GTP-bound Rheb proteins (Rheb-GTP) activate mTORC1 [35]. Very few studies have shown that, apart from insulin and IGF-I modulation, androgens stimulate Akt phosphorylation [36,37,38,39,40,41,42]. Mitogen-activated protein kinase (MEK1/2) phosphorylates mTORC1 by p90 ribosomal S6 kinase and Rs-dependent extracellular signal-regulated kinase (ERK1/2), while the former phosphorylates raptor at S719/722, and later phosphorylates raptor at S696, S863, and S6, thereby up-regulating mTORC1 activity [43,44]. Apart from growth factors, mTORC1 is also regulated by the energy status of the cell. The lack of cellular energy increases the AMP/ATP ratio, which activates (AMPK) AMP-dependent kinase [45]. Activated AMPK phosphorylates two residues of TSC2 viz. Thr1227 and Ser1345, and thus stimulates GAP activity of the complex through inhibition of Rheb, which in turn downregulates or inhibits mTORC1 activity (Figure 3) [46].

## 4. Downstream Effectors of mTORC1: S6K1 and 4EBP1

mTORC1 phosphorylates its downstream regulator (4EBP1, and S6K1) [47] through the interaction of raptor and TOR signalling (TOS) motif. The TOS motif is a five amino acid sequence located in C-terminus of 4E-BP1 (Phe-Glu-Met-Asp-Asp-I1e) and N-terminus of S6K1 (Phe-Asp-I1e-Leu), which is mandatory for mTORC1 to phosphorylate these proteins in vivo [48,49,50,51]. p70S6K1 is activated by MAPK, PDK1, and SAPK (stress-activated protein kinase). mTORC1 phosphorylates S6K1 at Thr389, which is important for its activation. mTOR inhibitors are known to diminish the Thr389 phosphorylation of the S6K1 [52,53]. Another downstream target of mTORC1 is 4E-BP1, which suppresses protein synthesis by binding to and inhibiting eIF4E (eukaryotic initiation factor 4E) [54,55]. When activated, mTORC1 promotes the dissociation of eIF4E from 4E-BP1, which enables free eIF4E to produce and initiate cap-dependent protein translation. Rapamycin inhibits mTORC1 and promotes dephosphorylation of 4EBP1, which limits denovo protein synthesis [56].

### 4.1. Eukaryotic Translation Initiation Factor 4E Binding Protein-1

mTOR and other kinases phosphorylate 4EBP1 at numerous serine/threonine sites in reaction to stimuli brought on by mitogens, growth factors, G-protein coupled agonists, and cytokines, which help in the dissociation of eIF4E from 4EBP1. This free eIF4E then binds to large scaffolding proteins, eIF4G, ATP-dependent RNA helicase eIF4A, and eIF4B, facilitating its cap-dependent protein translation [57,58]. The activation of mTOR signalling is often indicated by the phosphorylation of 4E-BP1. 4E-BP1 is a protein that contains seven different phosphorylation sites, namely Thr 37, Thr 46, Thr 70, Ser 65, Ser 83, Ser 101, and Ser, 112 [59]. Numerous experimental findings indicate that mTOR plays a direct role in phosphorylating 4EBP1 and activating eIF4E, which is simulated by various mitogenic signals [60]. The typical mTOR/4E-BP1 cascade may not be the only one that phosphorylates 4E-BP1, as numerous kinases have recently been proven to phosphorylate 4EBP1, either dependently or independently of mTOR [61]. Several signalling mechanisms have been proposed as potential kinases responsible for independent phosphorylation of 4EBP1 independent of mTOR pathway. These include extracellular signal-regulated kinase (ERK), glycogen synthase kinase-3 beta (GSK3β), prim-2 proto-oncogene, serine/threonine kinase (PIM2), ataxia telangiectasia mutated (ATM), cell division cycle protein 2/cyclin-dependent kinase-1 (CDC2/CDK1), p38 mitogen-activated protein kinases (p38MAPK), and leucine-rich repeat kinase 2 (LRRK2) signalling mechanisms. These findings suggest that mTOR inhibitors may not be absolutely effective in inactivating 4EBP1, which is activated alternatively by many other factors as discussed [62].

### 4.2. Ribosomal Protein S6 Kinase β-1 (S6K1)

The serine/threonine kinase S6K1 is another significant downstream target of mTOR. S6K1 is phosphorylated by activated mTOR on Thr389, which then phosphorylates S6K15, a component of the 40S ribosomal protein [63]. The ribosomal protein S6K Beta 1 (RPS6KB1) gene encodes two isoforms, p70S6K1 and p85S6K1 [64]. This mTOR-S6K1 signalling axis regulates important cellular functions like cell proliferation, metabolism, protein and lipid synthesis, translation, and transcription [64]. Additionally, this axis is responsible for adipocyte metabolism, learning, aging, memory, growth and development, while also controlling insulin sensitivity, glucose homeostasis, and other processes [65]. As a result, S6K1 is thought to be involved in critical functions in regulating cellular physiology [65]. Any disruption in this axis has negative consequences and can lead to the development of serious diseases ranging from metabolic disorders to cancer [64]. As a result, this network has remained a prime focus for different therapies used to treat different pathologies over the years. However, therapeutic interventions targeting mTOR in various cancers, notably renal and breast carcinomas, have resulted in a significant number of relapses due to the presence of feedback mechanisms in the signalling pathway [63]. Overcoming the resistance developed to mTOR inhibitors and other chemotherapeutic agents after chronic exposure to these drugs remains one of the key challenges that the scientific community is facing today.

## 5. Structure and Regulation of mTORC2

mTORC2 consists of four primary subunits, namely mTOR, RICTOR, mSIN1, and mLST8. Within mTORC1, the essential core subunit is regulatory-associated protein of mTOR (RAPTOR), while mTORC2 possesses distinct core subunits, namely rapamycin-insensitive companion of mTOR (RICTOR) and mammalian stress-activated Map kinase-interacting 1 (mSIN1). While numerous substrates of mTORC1 have been identified, mTORC2 predominantly phosphorylates AGC kinases, such as Akt (also referred to as protein kinase B, PKB), serum- and glucocorticoid-induced kinase 1 (SGK1), and members of the protein kinase C (PKC) family at their hydrophobic motif (HM) and turn motif (TM) [66]. 

The regulatory mechanisms of mTORC2 activity are lesser known than that of mTORC1. Recent studies suggest that mTORC2 is also regulated by several mechanisms. The platelet-derived growth factor, insulin like growth factor 1 receptor and epidermal growth factor receptor regulates its activity through PI3K signalling pathway, as PI3K helps binding of PIP3 with PH domain of mSin1, which restricts the kinase activity of mTOR, thereby activating mTORC2 and recruits it to the plasma membrane [66,67]. Growth factors stimulate phosphorylation of PI (4,5) P2 to produce phosphatidylinositol 3,4,5-triphosphate at plasma membrane by PI3K, this PIP3 has been proposed to directly activate mTORC2 [68]. Two models, which suggests the activation of mTORC2 are: PIP3 dependent activation, where mTORC2 is localized to plasma membrane, and other is that PIP3 brings conformational change in the mTORC2 leading to increase in its activity. However, emerging evidence suggests that mTORC2 permanently resides at plasma membrane and is constituently active [69].

It is well established that mTORC1 is responsive to both nutrition and growth factors, whereas mTORC2 is primarily controlled by growth factors [70]. Ample evidence suggests that amino acids lead to the activation of mTORC2. A reduction in glutamine catabolites caused by food restriction can activate mTORC2 and cause glutamine–fructose-6-phosphate amidotransferase 1 expression to rise (GFAT1) [71]. 

While PI3K regulation is the primary focus of most studies on mTORC2, recent research has unveiled the involvement of other signalling pathways in fine tuning mTORC2 activity. These include AMP-activated protein kinase (AMPK), Wnt signalling, and feedback control between mTORC1 and mTORC2.

AMP-activated protein kinase (AMPK) inhibits mTORC1 either by phosphorylating RAPTOR, or increasing the GTPase-activating protein (GAP) activity of TSC complex towards the GTPase Ras homology enriched in brain (RHEB) by phosphorylating TSC2, and hence indirectly activating mTORC2, which is responsible for the adaptation of low energy status of cells [46,72,73]. AMPK phosphorylates both Rictor and mTOR, which is primarily required and enough to increase the kinase activity of mTORC2. However, the molecular mechanism and phosphorylation sites, which regulate mTORC2 activity, are yet to be evaluated fully [74]. There is also a feedback control loops connecting mTORC1 and mTORC2. mTORC1 has a negative effect on mTORC2 through S6K1. S6K1, which is activated by mTORC1, causes inhibitory phosphorylation of IRS1 and reduces the amount of IRS1 protein. Inactivation of mTORC2 occurs as a result of the downregulation of insulin-PI3K signalling [75]. mTORC1 controls mTORC2 through an additional negative feedback loop involving growth factor-bound–receptor protein10 (Grb10) [76]. 

## 6. mTORC2 Effectors

mTORC2 is known to phosphorylate AGC kinases family proteins like Protein kinas A (Akt) and Protein Kinase C (PKC). It also phosphorylates glucocorticoid and serum-induced kinase-1 (SGK1) at their hydrophobic motif (HM) and turn motif (TM) [11].

The phosphorylation of HM motif of Akt at Ser473 [77] by mTORC2 is supposed to be a context-dependent event. This has been best illustrated in the study conducted by Estela Jacinto and his colleagues, where they have shown that Akt is incapable of phosphorylating FoxO1/3a, but predominantly phosphorylates other targets like TSC2 and GSK-3 in mSIN1 knockout mouse embryonic fibroblasts (MEFs) [17]. Similar to mTORC1, mTORC2 plays an important role in cell metabolism, regulating processes related to fatty acids, lipids, glucose, amino acid, and nucleotides [1]. It suppresses the activity of xCT (cysteine-glutamate antiporter), which is a solute carrier family 7 member 11 (SLC7A11) by phosphorylating it at xCT Ser26 [78].

## 7. Role of mTOR Signalling in Cancer at a Glance

Resistance to macrolide antibiotic, rapamycin in the yeast *Saccharomyces cerevisiae*, led to the discovery of the target of rapamycin (TOR). This eventually led to the discovery of the mammalian target of rapamycin (mTOR) in eukaryotes, which has the biochemical properties as that of the TOR [79]. The mTOR signalling pathway has a greater influence on basic critical cellular activities as its dysregulation disrupts normal physiological functioning in humans, leading to various diseases. It has been linked to various pathological conditions such as neuronal degeneration, obesity, type 2 diabetes mellitus, and cancer [9,80]. Both upstream and downstream effectors of mTORC1 play pivotal roles in the development of human cancers. Genetic mutations and amplifications are the two typical genetic alterations that cause protein molecules to become constitutively active. Hyper-activation of one of these upstream effectors, such as PI3K, Akt, or loss of phosphatase and TENs in homolog deleted on chromosome 10 (PTEN) molecules, triggers mTOR signalling cascade in cancer and plays a role in cellular proliferation, invasion, cytoskeleton rearrangement, metastasis, and cell survival and inhibits initiation of apoptosis and cellular autophagy.

Hanahan and Weinberg described that activation of mTOR signalling is the hallmark of a large number of cancers [81]. According to reports, more than 70% of malignancies have aberrantly overactivated mTOR pathways. It has been amply proven in recent years in cancer patients and animal models that mTOR malfunction promotes carcinogenesis [82,83]. Table 2 outlines how the activation of the mTOR pathway, either by oncogene stimulation or the loss of tumour suppressors can lead to the development of tumour angiogenesis and metastasis in various in-vitro cell lines and in-vivo mouse xenograft models [56].

The loss of PTEN function, receptor tyrosine kinase overexpression, or mutations in Akt and PI3K are not the only factors that can lead to mTOR activation. It can be activated through another mechanism that includes mutation and gene amplification [97]. COSMIC database analysis shows overexpression of mTOR in the skin (8.25%), urinary tract (8.33%), and ovary (9.77%) cancers, and downregulation in central nervous system cancers (13.06%). S2215Y [98] and R2605P are the two distinct point mutations that lead to mTOR activation in cells even under starved conditions as revealed in the COSMIC database. It has also been reported that meningeal cancers (18.63%) have the highest number of mutations in mTOR. The other cancer where mTOR is highly mutated is endometrioid carcinoma (12.63%) [23].

## 8. Autophagy, Apoptosis, and mTOR Signalling: A Connecting Link

Under normal and pathological circumstances, mTOR is implicated in the suppression of autophagy, which is otherwise triggered by nutrient stress and energy deprivation [99,100]. When autophagy is triggered, lysosomal degradation of cytosolic components occurs. Recent data have shown that mTORC1 phosphorylates UNC-5 like Autophagy Activating Kinase (ULK) 1/2, which results in its deactivation [101]. ULK1/2 phosphorylates ATG13 and FIP200 thereby initiating the autophagic processes [102,103]. mTORC1 also controls autophagy at the transcriptional level by regulating the localization of the transcription factor EB (TFEB), which is responsible for regulating genes involved in autophagy and lysosomes [104,105].

Up-regulation of mTOR1 inhibits glycogen synthase kinase (GSK-3) whose inhibition leads to the suppression of the caspases-3 signalling pathway, which ultimately reduces ROS generation. Decreased ROS generation leads to the inhibition of apoptosis [106,107].

In conclusion, it is inferred that the mTOR signalling pathway may contribute to cancer development by blocking autophagy and apoptotic pathways. 

## 9. mTOR Inhibitors in Cancer

As mTOR plays a crucial role in the development of tumours, mTOR inhibitors have the potential to be effective in various cancer treatments [108]. Rapamycin analogues (rapalogue) have received medical approval for the treatment of various cancers [109]. Additionally, other mTOR inhibitors with various modes of action have been developed. These rapalogues have been subclassified into three generations, some of which are presently undergoing clinical trials in a range of human cancers. 

### 9.1. First-Generation mTOR Inhibitors: Allosteric Inhibitors

#### Rapamycin and Analogues (Rapalogues)

**Figure 4:**
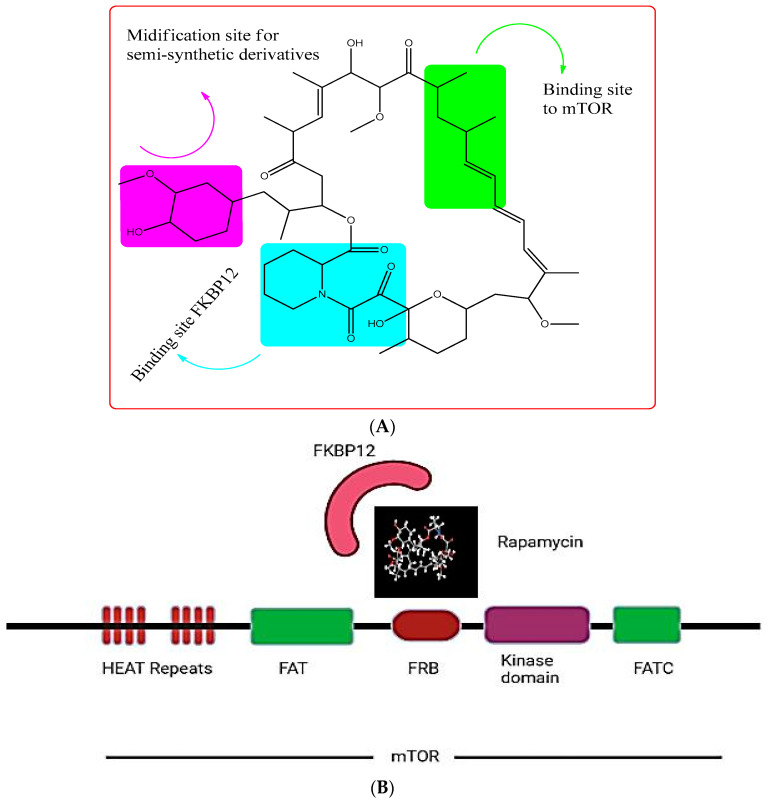
(**A**) showing the chemical structure of Rapamycin. (**B**) showing the rapamycin-mTOR interaction; rapamycin selectively binds and allosterictively inhibits mTORC1 at (FK506-binding protein) FKBP12.

In a soil sample from Easter Island, a fungus namely *Streptomyces hygroscopicus* was shown to generate the antifungal metabolite rapamycin (also known as “*Rapa Nu*” in the native language) (Figure 4A). It is a potent inhibitor of S6K1. Rapamycin specifically binds to the 12-kDa FK506-binding protein (FKBP12) and by doing so, it allosterically inhibits mTORC1. This leads to the inhibition of the intrinsic kinase activity of TOR, including to autophosphorylation, and prevents TOR from reaching its substrates (Figure 4B) [110]. As a result, drugs that exclusively target mTORC1, such as rapamycin, are likely to impede cancer metabolism and are seen as promising anti-cancer therapy agents. However, due to the poor solubility and pharmacokinetic profile of rapamycin have prompted the development of multiple rapamycin analogues (rapalogues). In 2007 and 2009, the Food and Drug Administration (FDA) permitted the use of two water-soluble rapamycin derivatives, temsirolimus and everolimus, respectively, to treat advanced renal malignant carcinoma (RCC).

However, the efficacy of rapamycin and rapalogues when used alone seems to be lower than anticipated [111]. Everolimus, an oral mTOR inhibitor, was first developed to suppress the immune system during solid-organ transplantation [112]. It inhibits both B and T cell proliferation and differentiation by blocking the cytokine-driven activation responses of these cells [113,114]. Its potential to act as an antiproliferative agent in various in vitro and in vivo studies led to its approval by FDA for different tumours. Its efficacy in treating these malignancies prompted the scientific world to investigate its impact on cancers having dysregulation of the critical PI3K/Akt/mTOR pathway [115,116]. The drug is known to inhibit HLA-I-stimulated cellular proliferation through the ERK1/2 mTORC1 signalling cascade. Despite the enormous therapeutic potential of mTOR inhibition alone, rapamycin and its derivatives (rapalogues) have limited effectiveness against specific substrates and can activate multiple negative oncogenic feedback loops. This has led to the development of new strategies to overcome these limitations. One approach was to combine rapalogues with other known inhibitors. Table 3 outlines the cellular effects of everolimus.

Temsirolimus, another rapalogue, has been extensively studied for its potential use in treating blood-related cancers. In clinical settings, it has been administered in combination with other medications. Patients with B-cell non-Hodgkin lymphoma and multiple myeloma who have received other treatments have been evaluated by using a combination of temsirolimus and bortezomib [117,118]. In both studies, participants were given intravenous temsirolimus (25 mg) once a week (on first day of week for 4 weeks), along with intravenous bortezomib (1.6 mg/m^2^) once a week (on first day of week for 4 weeks), with a treatment duration of 35 days. Around 33% of multiple myeloma patients showed a partial or better response to the treatment. A phase I clinical study has demonstrated that the addition of temsirolimus to rituximab and bendamustine resulted in promising outcomes in terms of both effectiveness and safety profile for patients with mantle cell lymphoma (MCL) and relapsed/refractory cancers [118,119].

### 9.2. Second-Generation mTOR Inhibitors: mTOR Kinase Inhibitors

In addition to rapalogues, second-generation mTOR inhibitors have generated mTOR to combat cancer in a better way, with one class inhibiting selectively both mTORC1 and mTORC2 without having any effect on other kinases. The other class of these second-generation mTOR inhibitors has shown the capability of inhibiting both mTOR and PI3K (dual inhibitors) [120,121]. As they can inhibit mTOR, PI3K, and Akt, they have the advantage of overcoming feedback loops [122]. A team of researchers who studied two mTOR inhibitors, namely PP242 and PP30, concluded that these inhibitors possess a central pyrazolo [3,4-d]pyrimidine ring with a C4 amino group, distinct heterocyclic substituents, and an N1 isopropyl substituent on C3. Both inhibitors competitively targeted mTORC1 and mTORC2 through ATP binding, exerting more significant effects on cell cycle regulation, cell growth, and proliferation, as well as cap-dependent translation when compared to the conventional inhibitor rapamycin [123]. Table 4 summarizes the second-generation mTOR inhibitors being tested at different phases of clinical trials.

### 9.3. Third-Generation mTOR Inhibitor: RapaLink

Since mTOR signalling pathway has an important role in normal cellular function, a complete blockade might have catastrophic effects [132]. Additionally, autophagy gets initiated by the blockade of mTORC1, thus promoting cancer cell survival as seen with the use of AZD8055 [133]. To solve this problem, Rodrick-Outmezguine developed a molecule by linking binding sites rapamycin and INK-128 leading to the generation of a dual mTOR inhibitor called Rapalink [134]. Its property of using the binding sites of both first- and second-generation inhibitors makes it a unique molecule to inhibit and be effective against drug-resistant mTOR mutants that have shown resistance to mTOR kinase inhibitors (TORKi). RapaLink-1 has been found more potent and effective in inhibiting mTOR than Rapamycin and second-generation mTOR inhibitors as evidenced by the treatment of RapaLink-1 in U87MG and LN229 cells. It has also been reported that FKBP12 bound to RapaLink-1 enhances the accumulation of Rapalink-1 molecules inside the cell, and thus makes it a potential drug to inhibit cancer-associated activating mTOR mutant [134,135]. A study conducted by Kazuki and collegues, 2020, reported that Rapalink-1 showed a better response in sunitinib-sensitive and sunitinib-resistant renal cell carcinoma (RCC) than temsirolimus. It has also been stated that rapalink-1 not only inhibits PI3K/AKT/mTOR but has a significant effect on ErbB (erythroblastic leukemia viral oncogene) signalling and ABC transporters [136].

It has been observed that MCF-7 cells treated with Rapalink did not develop resistance to chemotherapeutic drugs. However, significant resistance was observed after three months of treatment with either first- or second-generation mTOR inhibitors [109].

## 10. Combination Therapies

mTOR is the major signalling pathway involved in the development of resistance to already-existing cancer therapies. In recent years cancer biologists have shifted the focus to developing mTOR-based combination therapies. Positive feedback in PI3K/mTOR signalling pathway has limited the clinical effects of mTOR inhibitors through the activation of AKT by activation of downstream target and nuclear factor kappa B (Nf-κB), which helps in the accumulation of PIP3 and, hence continuous, AKT activation [137]. Built on these assumptions, that simultaneous inhibition of various signalling pathways together will minimize the incidences/chances of resistance, combining mTOR inhibitors with other drugs is now being explored. Numerous clinical trials are being conducted to evaluate the effectiveness of mTOR inhibitors in combination with other targeted therapies or chemotherapeutic drugs. To develop a successful combination therapy, issues related to toxicity and reliable biomarkers are important parameters to be kept in mind while selecting a patient to treat. As a result, a combination therapy of everolimus and exemestance is approved for human epidermal growth factor receptor 2 (HER2)-negative/Estrogen receptor (ER)-positive breast cancer [138]. Another study conducted by Mortzer and colleagues reported that everolimus, when combined with lenvatinib (vascular endothelial growth factor inhibitor), is found to be more effective in metastatic renal cell carcinoma than when used alone [139]. Also phase-II clinical trial of a combination of letrolzole, an aromatase inhibitor, and the rapamycin analogue, everolimus, has shown promising results in oestrogen receptor-positive ovarian cancer, achieving 12-week progressive free survival in 47% of patients [140]. Table 5 summarizes the mTOR-based combination therapies that are being explored in clinical trials. Overall, these studies using mTOR-based combination therapies have suggested that using mTOR inhibitors with other anti-cancer agents to overcome the limitations facing these regimens when using alone. 

Since insulin-like growth factor 1 and insulin receptor (IGF-IR) signalling could potentially lead to resistance against mTORC1 inhibitors, a clinical trial was carried out to evaluate the effectiveness of combining cixutumumab, a humanized monoclonal antibody targeting IGF-1R, with temsirolimus. The outcome of the trail was that the combination therapy exhibits better clinical response in patients diagnosed with sarcoma and adrenocortical carcinoma than when temsirolimus was used alone [141].

In another clinical trial, CTRI/2018/05/014178, conducted by Timothy Crook and co-workers in 2020, it was demonstrated that mTOR-based drug combination improved treatment outcomes, like response rate and disease control rate [142]. 

Despite attempts to use combination therapies, favourable outcomes are not consistently achieved. This was evident in a clinical trial where the combination of everolimus and the epidermal growth factor receptor (EGFR) inhibitor gefitinib showed limited effectiveness in reducing tumour activity in patients diagnosed with metastatic castration-resistant prostate cancer [143]. Similarly in patients with advanced solid tumours, the combination of pimasertib and voxtalisib exhibited inadequate long-term tolerability and restricted effectiveness in reducing tumour activity [144].

The combinations of mTOR inhibitors with other targeted agents or cytotoxics have been shown to delay the emergence of resistance to these agents. They have demonstrated the potential anticancer activity against several types of cancers including hormone positive and HER2- negative breast cancers, whose treatment otherwise is challenge [145]. 

**Figure 5:**
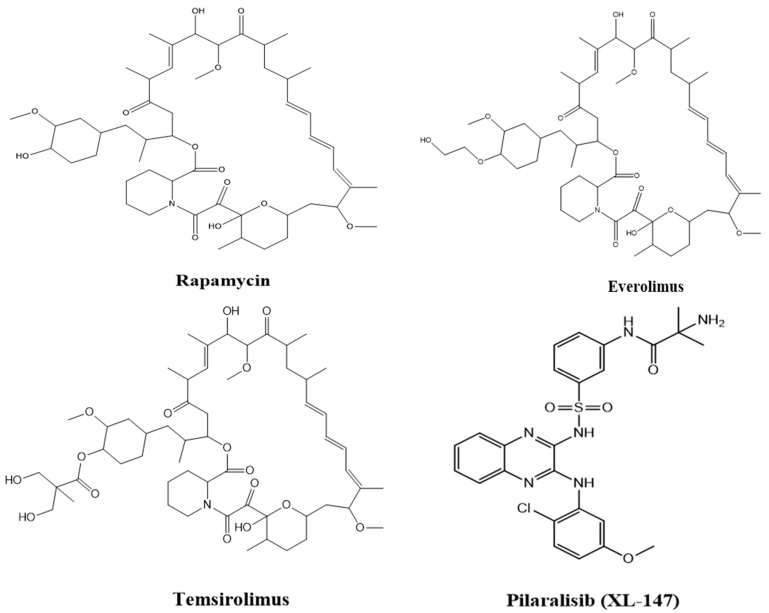
Structures of various mTOR inhibitors.

**Figure 6:**
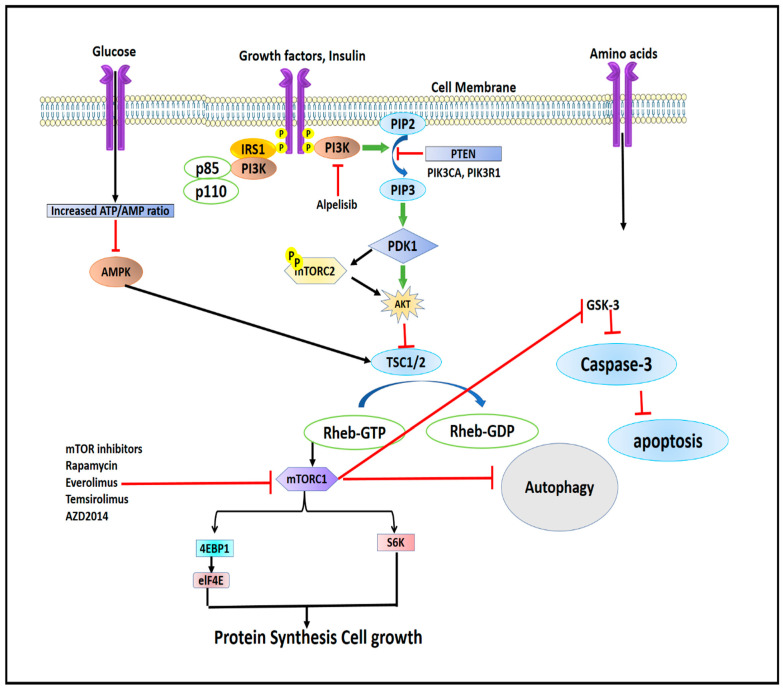
Illustrating the mTOR signalling and effect of its inhibitors.

Figure 5 shows the structures of some of the mTOR inhibitors, while Figure 6 illustrates the mTOR signalling and the effect of its inhibitors.

**Table 5:** Summary of the mTOR-based drug combinations investigated in various cancers.

mTOR Inhibitor	Combined with Drug	Tumour Applied and Type of Study	Outcome	Ref.
Everolimus	Trametinib (kinase inhibitor)	Advanced solid tumours/ Phase 1B NCT00955773	Among 67 patients, 5 patients (7%) achieved partial response (PR) to treatment and 21 (31%) displayed stable disease (SD)	[146]
Everolimus	Lenvatinib (multiple receptor kinase inhibitor)	RCC/Phase-II NCT01136733	Survival rate increased when used in combination	[139]
Everolimus	Carboplatin and paclitaxel	LCNEC/ Phase II NCT01317615	Improvement in overall response rate and tumour regression, combination is effective and well tolerated than using drug alone	[147]
Rapamycin	Entinostat (benzamide histone deacetylase inhibitor)	General cancers/ In vitro	This led to the halting of the cell cycle and the start of programmed cell death (apoptosis), it promotes MYC degradation	[148]
Rapamycin	AR inhibitor enzalutamide	HCC/ In vitro and In vivo	Enalutamide and rapamycin together yielded stronger anti-HCC activity than each drug alone in vitro and in vivo. Also, combination exhibited more potent antitumour activity in the xenograft tumour model than cultured cancer cells, causing elevated apoptotic cell death and tumour regression	[149]
Rapamycin	STX-0119	Glioblastoma/ In vitro	Combining of two drugs had significant growth inhibitory effect against the TMZ-R U87 cell line. IC50 decreased to 11.3μM (drug combination) from 78 μM (STX-0119) and 30.5 μM (rapamycin)	[150]

AR: androgen receptor, HCC: hepatic cell carcinoma, LCNEC: large-cell neuroendocrine carcinoma, RCC: renal cell carcinoma.

## 11. Adverse Events Related to the Use of mTOR Inhibitors

The side effects of modern targeted therapies differ from those observed with traditional chemotherapy. Mammalian target of rapamycin (mTOR) inhibitors have attained significant attention in the field of oncology and exhibit a wide range of toxic effects like headache, mucositis, rashes, and metabolic toxicities including hyperglycemia, hyperlipidemia, and hypophosphatemia. These adverse effects can be mild to severe, which require medical attention [151].

Skin toxicity, usually on face and neck, occurs with the use of mTOR inhibitors. The reported rate of skin rashes with the use of everolimus is 25–50%, temsirolimus 47–76%, and ridaforolimus 48–66% [151,152,153,154].

Development of adverse reactions or events (AR/AE) with the use of mTOR inhibitors requires attention, and it is important to understand their underlying mechanisms for managing such AEs [154]. Pneumonitis [155], metabolic adverse events like hyperglycemia (13–50%) and hyperlipidemia (12%) with the use of everolimus and 11% with the use of temsirolimus [156]. The incidence of mTOR inhibitors associated stomatitis (mIAS) has been also reported in some studies [157]. The use of mTOR inhibitors has been associated with haematological adverse events such as thrombocytopenia and leucopenia/neutropenia and, hence, their use requires routine complete blood counts [158].

Fatigue, weakness, changes in taste perception, and diarrhoea are among the frequently observed adverse effects in clinical trials involving mTOR inhibitors [159].

The novel mTOR inhibitors also have been associated with bone marrow suppression. In a phase III clinical trial of temsirolimus, 45% of patients reported anaemia, and 20% of them had severe anaemia (grade 3–4) [152]. Similarly, in another phase III clinical trial with everolimus, it was revealed that 91% patients had anaemia, with 9% grade three, and 1% with grade four [160]. 

A meta-analysis conducted by Jian Xu and Deying Tian in 2014 included a total of 5436 patients with various solid tumours from 26 clinical trials. The study findings indicated the following rates of hematologic toxicities associated with mTOR inhibitors: anaemia in 38.8%, with 7.5% of patients experiencing severe anaemia; leucopenia in 19.6%, with 1.8% of patients experiencing severe leucopenia; and neutropenia in 14.9%, with 5.6% of patients experiencing severe neutropenia [161]. Table 6 summarizes the AE/AR associated with the use of mTOR inhibitors in various cancers.

## 12. mTOR Inhibitors in Combination with Chimeric Antigen Receptor Treatment (CAR-T) Therapy and Immune Check Point Inhibitor (ICI) Therapy

Managing advanced and metastatic cancers poses a formidable challenge, which is why combination therapies are employed to effectively address the underlying cause of the issue. Numerous combination therapies have been investigated so far, and among the recently explored approaches in the treatment of various solid tumours is the combination of CAR-T, ICI, and mTOR-based therapies. A study conducted by Zhigang Nain and co-workers reported that by utilizing rapamycin as a pretreatment to reduce mTORC1 activity, the ability of CAR-T cells to penetrate the bone marrow was enhanced, resulting in an increased elimination of acute myeloid leukaemia (AML) cells in the bone marrow of leukemia xenograft mouse models [162].

Similarly, the combination of mTOR inhibitors with immunotherapies like ICI has been the subject of numerous preclinical studies, which have underscored the potential anticancer advantages of such combinations. In their study, Moore and coworkers revealed that combining rapamycin and anti-PD-L1, a monoclonal antibody (mAb), resulted in increase in survival rate of mice with murine oral cancer cell line immunogenic (MOC1) tumour when compared to the monotherapy [163]. 

In a similar manner to rapamycin, the targeting of mTOR using kinase inhibitors enhances the anti-cancer effects of checkpoint inhibitors. In fact, the combination of the mTORC1/mTORC2 inhibitor vistusertib with anti-CTLA-4, anti-PD-L1, and anti-PD-1, demonstrated a significant decrease in the growth of MC38 or CT-26 (murine colorectal cancer cell lines) tumours compared to individual therapies [164].

Collectively, these studies provide ample evidence that combining mTOR inhibitors with immune checkpoint modulators and CAR-T therapy have advantages over monotherapy in the context of cancer treatment.

## 13. Clinical Application of mTOR Inhibitors against Various Cancers

Due to the observed anti-cancer effectiveness of mTOR inhibitors in preclinical studies, either as standalone treatments or in combination with chemotherapy, radiotherapy, and targeted therapy, numerous clinical trials have been completed or are currently underway to assess the efficacy of mTOR inhibitors in the treatment of various types of human cancers. Multiple mTOR inhibitors have received approval for the treatment of human cancers. Many more studies/clinical trials are currently underway to assess the efficacy of mTOR inhibitors [165]. The published data support that use of mTOR inhibitors against oesophageal cancer (everolimus, NCT00985192 by translational oncology research international, USA in 2009) [165], gastric cancer (everolimus, NCT00519324 by Novartis pharmaceuticals in 2009) [166], everolimus plus cisplatin, NCT00632268 by National Taiwan University Hospital in 2008 [167], hepatocellular carcinoma, pancreatic cancer, and colorectal cancer, where a number of mTOR inhibitors have been tested and found safe and effective against the disease. 

Based on the clinical data that have been published, the efficacy of using mTOR inhibitors alone against gastrointestinal cancers is restricted and might be due to the feedback regulation mechanisms, which impede the therapeutic impact of mTOR inhibitors. Consequently, the most promising approach for utilizing mTOR inhibitors in the future would involve combining them with other types of drugs such as radiation therapy, antibody drugs, and other cytotoxic drugs.

## 14. Conclusions

mTOR is the critical pathway in modulating cellular proliferation, apoptosis, and autophagy of cells by influencing transcription and protein synthesis through the integration of diverse extracellular and intracellular signal stimuli. mTORC1 and mTORC2 are two separate complexes, which are cross-talked with each other. Understanding the role of mTOR downstream regulators, 4E-BP1 and S6K1 is important in treating cancer at later stages because of their active role in cellular proliferation, protein synthesis, tumour angiogenesis, and metastasis. Since major developments in understanding this crucial signalling pathway are primarily focused on the use of rapamycin or rapalogues, which primarily inhibit mTORC1 activity, novel dual PI3K/mTOR inhibitors, and selective mTORC1/mTORC2 inhibitors are being evaluated at clinical levels to overcome the challenges like resistance and feedback effects of already known mTOR inhibitors. Also, several studies provide ample evidence that combining mTOR inhibitors with immune checkpoint modulators and CAR-T therapy have advantage over monotherapy in the context of cancer treatment. However, the toxicity and adverse effects of these mTOR inhibitors make it difficult to treat cancer, which has significant negative effects on the patients. As a result, it is important to conduct more clinical trials to understand the underlying mechanisms associated with the use of mTOR inhibitors so that they can be used to counteract these toxicities for their safe and effective treatments.

## Figures and Tables

**Table 2:** Tumour-associated genes and cancer.

Tumour Promoter Genes	Associated Cancers	Reference
S6K1	In lung and ovarian malignancies, expression of S6K1 is high, while in breast, kidney, and hepatocellular carcinomas, its high expression is associated with a bad prognosis.	[84,85,86]
PI3K	In ovarian, gastrointestinal, breast, and prostate cancers, high PI3K activity is linked to cellular transformation and tumour growth.	[87,88,89]
Akt	Subgroups of human malignancies, like breast and ovarian tumours, have increased levels of Akt.	[87]
Rheb	Numerous tumour cells have overexpressed Rheb levels. Increased Rheb level is related to poor prognosis of breast, head, and neck malignancies.	[90]
eIF4e	The overexpression of eIF4E, whether in vitro or in vivo, can cause changes in cells. Furthermore, eIF4E is found to be overexpressed in numerous types of cancers, such as nonlymphomas, Hodgkin’s acute and myelogenous leukemia, as well as colon, head, breast, and neck malignancies.	[84,90]
4E-BP1	4EBP1 expression has been linked to a poor prognosis. Also, its phosphorylation has been linked with chemoresistance in ovarian cancer	[91,92]
**Tumour suppressor genes**		
TSC1/TSC2	The occurrence of mutations in the TSC can result in the formation of hamartomas in several organs.	[93]
PTEN	The function of PTEN is frequently lost in varied cancers like renal, breast and prostate.	[88,94]
LKB1	People who have genetic changes in the LKB1 gene can develop a condition called Peutz–Jeghers syndrome, which is characterized by the growth of abnormal tissue called hamartomas in the gastrointestinal tract	[95,96]

**Table 3:** The impact of everolimus treatment on cell functioning.

Down-Regulation	Up-Regulation	Cellular Effects
4E-BP1 and S6K1	LC3B and beclin1	
CDK2	FOXO family	
PPARγ		Increases response to radiation therapy
HIF-Iα		
PPARα		Increases response to radiation therapy
PGC1α		
Akt	BAD	
Myc		
Cyclin A and D1	GSK3	G_0_/G_1_ cell cycle arrest
Myc	CDK inhibitors p21 ^cip1^ and p27 ^kip1^	Decreased cellular proliferation
VEGF		

**Table 4:** Summary of second-generation mTOR inhibitors.

Compound/Molecule	Company Producing	Generic Name Given	The Phase of the Study	Condition Used for	Mechanism of Action	Clinical Outcome	Clinical Trail Registration Number	Ref.
GDC-0980	Genentech	Apitolisib	I and II	Lymphomas, renal, and breast cancer	Inhibits both mTOR and PI3K	Less effective than eveloremus	NCT01442090	[124]
XL765	Exelixis/Sanofi-Aventis	Voxtalisib	I-II	Lung, Breast cancer	Inhibits both mTOR and PI3K	Has better safety profile	NCT01403636	[125]
Pf-05212384	Pfizer	Gedatolisisb	I-II	Colorectal and Breast cancer	Inhibits both mTOR and PI3K	Gedatolisib combination therapy showed an acceptable tolerability profile	NCT01920061	[126]
NVP-BEZ235	Novartis	Doctolisib	I-II	Sarcoma, leukemia, prostate, and renal cancer	Dual PI3K/mTOR inhibitor	Comibation of NVP-BEZ235 with evelorimous resulted in increase steady state pharmacokinetics of evelorimous	NCT01508104	[127]
TAK-228	Intellikine	Sapanisertib	I-II	Lymphomas, Advanced solid tumours	Selective mTORC1/2 inhibitor	It demonstrated improved safety profile	NCT01058707	[128]
CC-223	Celgene	Pilaralisib	I-II	Advanced solid tumours	Selective mTORC1/2 inhibitor	High durable tumour regression, improved safety profile, and high durable response	NCT01177397	[129]
AZD8055	AstraZeneca		I-II	Advanced solid tumours, Lymphomas	Selective mTORC1/2 inhibitor	Better safety profle than different rapalogues but showed an elevated transaminase levels, now AZD2014 is now being developed, which has reported no rise in transaminase levels	NCT00731263	[130]
XL147	Exelixis/Sanofi-Aventis		I-II	Lung, Breast, and Glioblastoma	Dual PI3K/mTOR inhibitor	Complete or partain response, with better sfaety profile	NCT01240460	[131]

**Table 6:** Summary of AR/AE with the use of some of the mTOR inhibitors.

Type of Toxicity	Temsirolimus [152]		Everolimus [160]		Ridaforolimus [153]	
	All Grades (%)	Grade ¾ (%)	All Grades (%)	Grade ¾ (%)	All Grades (%)	Grade ¾ (%)
Anemia	29–45	9–20	91–92	9–13	53	0
Skin rashes	47–76	4	25–29	<1	48–66	2–3
Hyperglycemia	26–89	17–16	50–57	12–15	22	6–13
Hypophosphatemia	13–49	13–18	32–37	4–6	23	15
Mucositis	20–75	1–4	40–44	3–4	45–78	15–16
Fatigue	38–51	8–11	31–38	4–5	20–76	3–4

## Data Availability

Not applicable.
